# Using estrogen and progesterone to treat premenstrual dysphoric disorder, postnatal depression and menopausal depression

**DOI:** 10.3389/fphar.2025.1528544

**Published:** 2025-02-20

**Authors:** Eveline Mu, Lauren Chiu, Jayashri Kulkarni

**Affiliations:** Department of Psychiatry, HER Centre Australia, The School of Translational Medicine, Monash University, Melbourne, VIC, Australia

**Keywords:** premenstrual dysphoric disorder, postnatal depression, menopausal depression, estrogen, progesterone, gonadal hormones, women's mental health

## Abstract

Female gonadal hormones, particularly estrogen and progesterone, are not only central to reproductive health but also play a crucial role in regulating mood, cognition, and overall brain health. These hormones have a significant impact on the central nervous system, influencing key processes such as neurotransmission, neuroplasticity, and brain development. Increasing evidence shows that hormonal fluctuations contribute to the onset and progression of mental health disorders that disproportionately affect women, particularly premenstrual dysphoric disorder (PMDD), postnatal depression (PND), and menopausal depression. This paper explores the current evidence regarding the neurobiological effects of female hormones on the brain and discusses the therapeutic approaches in conditions such as PMDD, PND, and menopausal depression.

## 1 Introduction

Gonadal hormones–estrogen and progesterone–play critical roles in maintaining mental health across the lifespan. Beyond their well-known function in regulating reproductive processes, these hormones are neuroactive steroids that influence various brain functions, including mood regulation, cognitive function, and emotional processing (Rubinow and Schmidt, 2002; van Wingen et al., 2011). This relationship between gonadal hormones and brain function has been studied extensively, with clear evidence showing that fluctuations in these hormones can significantly affect mental wellbeing, particularly in women who experience more frequent and pronounced hormonal changes (Del Río et al., 2018; Kulkarni, 2023).

Estrogen is widely regarded as a neuroprotective hormone (Dubal and Wise, 2002; Kulkarni, 2011). It modulates synaptic plasticity, promotes neurogenesis, and has direct effects on key neurotransmitter systems, including serotonin, dopamine, and gamma-aminobutyric acid (GABA) (Bendis et al., 2024). Specifically, estrogen influences the serotonergic system by regulating the expression and activity of serotonin transporters and receptors, which are critical for mood regulation (Joffe and Cohen, 1998; Wharton et al., 2012). For example, serotonin transporter levels increase in response to estrogen, enhancing serotonin reuptake efficiency and stabilising mood (McEwen et al., 1997). Additionally, estrogen has antioxidant properties and helps maintain mitochondrial function, protecting neurons from oxidative stress and energy deficits, both of which are implicated in neurodegenerative conditions (Lejri et al., 2018).

Progesterone and its metabolite, allopregnanolone (ALLO), exert their influence primarily through the GABAergic system, the major inhibitory neurotransmitter system in the brain. ALLO, in particular, enhances GABA-A receptor activity, enhancing inhibitory tone (Belelli and Lambert, 2005) and producing anxiolytic and antidepressant effects (Diviccaro et al., 2022). However, cyclical changes in progesterone and ALLO levels, such as those occurring in the luteal phase of the menstrual cycle or *postpartum* period, can destabilise this system (Gilfarb and Leuner, 2022). This destabilisation is hypothesised to arise from shifts in receptor sensitivity or downstream signalling pathways, leading to heightened vulnerability to mood disorders in susceptible individuals (McEwen, 2003).

**FIGURE 1:**
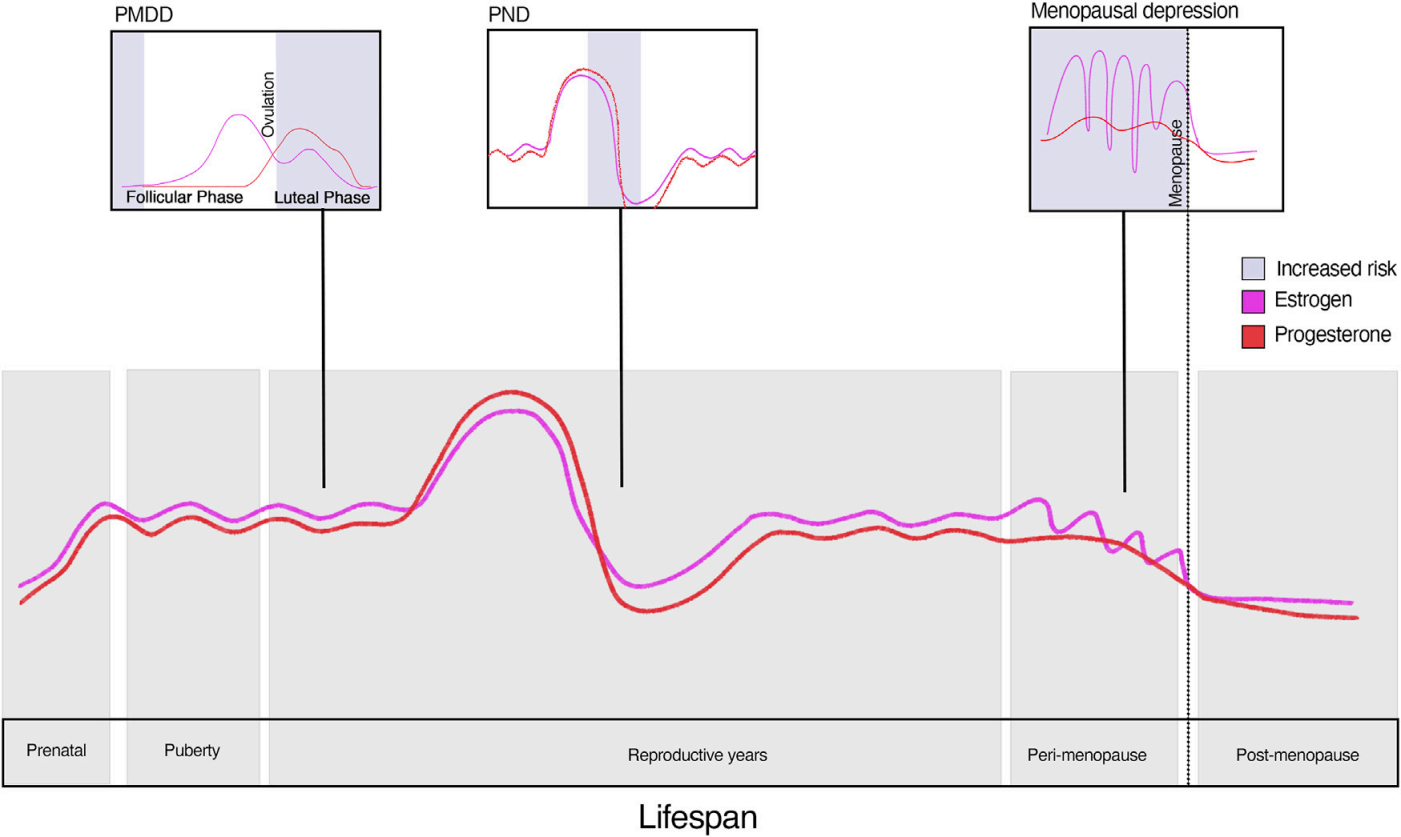
Risk of depression across the lifespan.

The hormonal changes that occur throughout a woman’s life–during the menstrual cycle, pregnancy, *postpartum* period, and menopause–are strongly linked to mood disorders in some women, suggesting a direct link between hormonal fluctuations and mental health (Figure 1). The heightened prevalence of disorders such as depression and anxiety in women across the lifespan highlights the importance of understanding the role of these hormones in brain function. This review aims to examine the efficacy of current hormone treatments for premenstrual dysphoric disorder (PMDD), postnatal depression (PND), and menopausal depression, synthesising findings from relevant studies and highlighting their clinical implications.

## 2 Premenstrual dysphoric disorder (PMDD)

PMDD is a severe form of cyclical depression characterised by intense mood swings, irritability, cognitive challenges, depression, and anxiety during the luteal phase of the menstrual cycle. Ppremenstrual syndrome is a milder form of luteal phase-related physical symptoms (fluid retention, bloating, headaches) and some irritability. Ppremenstrual exacerbation refers to the exacerbation of existing mental illnesses during the late luteal phase and improving with menses.

PMDD affects an estimated 3.2% of women of reproductive age (Reilly et al., 2024), using a strict definition. The condition is probably much more prevalent when irregular menstrual cycles and broader definitions of mental ill health symptoms are included. The timing of PMDD symptoms - with an onset in the late-luteal phase and resolution with menstrual bleeding - suggests that gonadal hormonal fluctuations play a significant role. Women with PMDD are thought to have a heightened sensitivity to these hormone changes in the central nervous system (Schmidt et al., 1998; Schmidt et al., 2017).

An increasing body of research suggests that ALLO may be involved in the pathophysiology of PMDD (Martinez et al., 2016; Hantsoo and Epperson, 2020; Gao et al., 2023). In PMDD, fluctuations in ALLO levels during the menstrual cycle, particularly the luteal phase, appear to contribute to mood symptoms. Some studies have shown decreased peripheral ALLO during the luteal phase of affected women, hence disrupting emotional regulation in those sensitive to these variations (Rapkin et al., 1997; Klatzkin et al., 2006; Schiller et al., 2014; Hantsoo and Epperson, 2020).

The discovery of the Extra Sex Combs/Enhancer of Zeste (ESC/E(Z)) gene network, found to be altered in over 50% of women with PMDD (Dubey et al., 2017), further adds to the neurobiological underpinning of this condition. The ESC/E(Z) gene network regulates gene expression in response to gonadal hormones. Dysregulation within this network is believed to increase sensitivity to fluctuations in gonadal hormones, such as estrogen and progesterone (Dubey et al., 2017). The altered gene complex is also presumed to interact with environmental factors, such as emotional, physical or sexual trauma, to exacerbate symptoms (Dubey et al., 2017). This interaction suggests that epigenetic changes mediated by hormonal fluctuations may underline the heightened vulnerability to PMDD, offering a potential avenue for targeted therapeutic interventions.

Interestingly, despite their heightened sensitivity, women with PMDD show no significant differences in standard gonadal hormone levels compared to those without symptoms (Hantsoo and Epperson, 2015). This increased sensitivity to otherwise typical hormonal shifts, appears to be a central factor in the development of PMDD (Backstrom et al., 2011; MacKenzie and Maguire, 2014; Schweizer-Schubert et al., 2021).

### 2.1 Hormone treatments for PMDD

The first-line treatments for PMDD are typically either antidepressant medications, commonly selective serotonin reuptake inhibitors (SSRIs), or oral contraceptives. Although less research has focused on serotonin-norepinephrine reuptake inhibitors (SNRIs), existing evidence indicates that venlafaxine (Hsiao and Liu, 2003; Lee et al., 2004) and duloxetine (Mazza et al., 2008; Ramos et al., 2009) can be effective for treating PMDD. Clomipramine, a tricyclic antidepressant with strong serotonin reuptake inhibition, has also been shown to alleviate the emotional and physical symptoms of PMDD (Sundblad et al., 1993). SNRIs are typically a second-line treatment when tolerability issues limit the use of SSRIs, while clomipramine, which is not US Food and Drug Administration (FDA) approved for PMDD, is prescribed off-label. Antidepressants, whether administered continuously or intermittently, may provide partial symptom relief; however, they do not directly address the underlying hormonal sensitivity thought to drive PMDD. Combined oral contraceptive pills (COCPs) are also frequently prescribed as an initial treatment option to suppress ovarian activity, as they induce an anovulatory cycle (Table 1).

**TABLE 1:** Summary of hormone treatment options for PMDD.

Drug type	Drug name	Dosage	Use recommendation	Findings/comments
Combined oral contraceptive pill (COCP)	Drospirenone with ethinylestradiol (Yasmin)	Drospirenone 3 mg, ethinylestradiol 30 µg	Take from Day 1 of menstrual cycle and continue for 24 consecutive days, then inert placebo pills on last 4 days	Antiandrogenic progestins may improve irritability (Kelderhouse and Taylor, 2013); and effectively reduce PMDD symptoms compared to placebo (Pearlstein et al., 2005)
	Nomegestrol acetate with 17-beta estradiol (Zoely)	Nomegestrol acetate 2.5 mg, 17-beta estradiol 1.5 mg	Take from Day 1 of menstrual cycle and continue for 21 consecutive days, then inert placebo pills on last 7 days	Significant premenstrual improvements in dysmenorrhoea, fluid retention, sadness, concentration difficulties, and behavioural changes compared to Yasmin (Witjes et al., 2015)
	Levonorgestrel with ethinylestradiol	Levonorgestrel 90 μg, ethinylestradiol 20 µg	Continuous dosing regimen without hormone-free interval	Poor evidence for alleviating PMDD symptoms (Freeman et al., 2012)
Estradiol	Estrogen	Transdermal patch 100–200 µg twice weekly or gel 0.75–1.5 mg daily	Take in combination with cyclical progestogen (1–5 mg oral norethisterone) continuously or during the luteal phase	Very low-quality evidence to support the use of continuous estrogen with progestogen, or luteal-phase unopposed oral estrogen (Naheed et al., 2017)
Progesterone	Micronised progesterone	100–200 mg daily	Take during the luteal phase to manage PMS symptoms	Lower risk of androgenic side effects (Memi et al., 2024)
Selective progesterone receptor modulator	Mifepristone	5 mg alternate days	Low-dose administration in the luteal phase	No significant improvement in symptoms of severe PMS (Chan et al., 1994)
Second-generation selective progesterone receptor modulator	Ulipristal acetate	5 mg daily	For PMDD	Significant symptom improvement compared to placebo (Comasco et al., 2021)
Synthetic allopregnanolone	Brexanolone	Infusion 30 μg/kg per h (0–4 h); 60 μg/kg per h (4–24 h); 90 μg/kg per h (24–52 h); 60 μg/kg per h (52–56 h); 30 μg/kg per h (56–60 h)	Single continuous intravenous infusion for 60 h	May be a novel treatment option for PMDD given similar symptoms with PND(Kanes et al., 2017; Meltzer-Brody et al., 2018)
Isoallopregnanolone, GABA-A antagonist	Sepranolone	10 or 16 mg subcutaneously every second day	Administer during luteal phase	No longer in production; reduced PMDD symptoms by 75% compared to placebo (Bixo et al., 2017)
	Dutasteride	2.5 mg daily	Used to treat symptoms of benign prostatic hyperplasia. Off-label option for women with PMDD experiencing side effects or lacking benefits of SSRIs	Prevents the luteal phase increase in ALLO and reduces core PMDD symptoms such as irritability, sadness, anxiety, food cravings, and bloating compared to placebo (Martinez et al., 2016)
Gonadotropin-releasing hormone (GnRH) analogue	Zoladex	3.6 mg depot for 6 cycles	For severe cases of PMDD, limit to 6 months of use or use in combination with estradiol and progestogen or tibolone	Effective for reducing physical and emotional premenstrual symptoms (Wyatt et al., 2004)
	Leuprolide	3.75 mg depot once a month for 3 months		
	Buserelin	Intranasal spray 100 μg daily for 2 cycles, 2 × 2 cycles		

#### 2.1.1 Combined oral contraceptives (COCPs)

The effectiveness of COCPs in managing mood symptoms has mixed results. Some studies indicate that specific COCPs may exacerbate mood symptoms (Kulkarni, 2007; Skovlund et al., 2016), while others report neutral or positive effects on mood, suggesting that results may vary based on factors such as the population studied, COCP formulation, and dosing regimen (continuous versus intermittent use) (Mu and Kulkarni, 2022).

Recent research into COCPs containing antimineralocorticoid and antiandrogenic progestins, such as drospirenone, shows promising outcomes for managing PMDD (Kelly et al., 2010) (Table 1). COCPs containing antiandrogenic progestins may improve irritability—a key symptom of PMDD (Kelderhouse and Taylor, 2013). Early studies by Freeman et al. (2001) and Pearlstein et al. (2005) demonstrated that drospirenone (3 mg) combined with ethinylestradiol (30mcg), as in Yasmin™ (Bayer Healthcare) effectively reduced PMDD symptoms compared to placebo. A subsequent Cochrane review (Lopez et al., 2012) also suggested that COCPs with drospirenone (3 mg) may benefit PMDD symptom management, although a notable placebo effect was noted. Recent reviews affirm the positive outcomes of drospirenone (3 mg) combined with ethinylestradiol (30mcg) in treating PMDD, but emphasise the need for further research (De Berardis et al., 2022). In contrast, COCPs with levonorgestrel (90 mcg) and ethinylestradiol (20mcg) have not alleviated PMDD symptoms (Freeman et al., 2012).

A newer COCP, nomegestrol acetate (2.5 mg) and 17β-estradiol (1.5 mg) (Zoely™, Merck Sharp & Dohme), has shown promise for off-label treatment of PMDD-related mood symptoms (Table 1). This monophasic formulation includes 24 active pills followed by four placebo pills (2014). It contains a synthetic estrogen (17β-estradiol) that is structurally identical to endogenous estrogen, whereas most other COCPs contain ethinylestradiol. 17β-estradiol can cross the blood brain barrier, interact with serotonin receptors, regulate cerebral blood flow to the amygdala and dorsolateral prefrontal cortex, and many other areas of the brain, including important mood-control brainstem centres, all involved in depression (Rubinow and Girdler, 2011).

Nomegestrol, structurally similar to progesterone, provides strong antigonadotrophic and moderate antiandrogenic activity, with no effects on estrogenic, glucocorticoid or mineralocorticoid pathways. A pooled analysis by Witjes et al. (2015), incorporating studies by Mansour et al. (2011) and Westhoff et al. (2012), evaluated the effects of nomegestrol acetate/17β-estradiol versus drospirenone/ethinylestradiol on premenstrual and menstrual symptoms using the Menstrual Distress Questionnaire Form C (Moos R, 1968). Women taking nomegestrol acetate/17β-estradiol (Zoely ™) experienced significant improvements in dysmenorrhoea, fluid retention, sadness, concentration difficulties and behavioural changes during the premenstrual phase compared to those taking drospirenone/ethinylestradiol (Yasmin ™) (Witjes et al., 2015). Our recent pilot study supports the acceptability and effectiveness of nomegestrol acetate/17β-estradiol (Zoely ™) treatment for PMDD symptoms, with 74.5% of women tested, reporting a positive mood response (Robertson et al., 2021). Significant reductions in scores on the Depression, Anxiety, Stress Scales – 21, a widely used self-report instrument for assessing the severity of symptoms across these three domains (Henry and Crawford, 2005), were observed after treatment initiation. These findings suggest the potential efficacy of nomegestrol acetate/17β-estradiol in managing mood symptoms associated with PMDD. Further research is needed to validate the efficacy of this COCP and understand its mechanism of action.

#### 2.1.2 Estrogen

Transdermal estradiol, delivered via a patch or gel, in combination with cyclical progestogen, has been shown to effectively manage both the physical and psychological symptoms of PMDD (Table 1). These percutaneous formulations provide sufficient estradiol to suppress ovarian function. Clinical trials indicate that 17β-estradiol, when paired with cyclical progestogens, is effective in reducing severe premenstrual syndrome symptoms (Panay et al., 2001; Studd, 2012). However, recent systematic reviews have rated the quality of this evidence as low (Naheed et al., 2017). The tested dose of the transdermal patch has ranged from 100 to 200 µg twice weekly during the luteal phase, with lower doses generally being better tolerated (Naheed et al., 2017). However, the long-term adverse effects of these treatments remain insufficiently studied. When administering percutaneous estradiol, oral or vaginal progesterone is also prescribed to prevent endometrial hyperplasia.

#### 2.1.3 Progesterone

Micronised oral progesterone presents a potential treatment option for PMDD (Table 1), offering a lower risk of androgenic and other unwanted side effects compared to progestogens such as norethisterone and levonorgestrel (Memi et al., 2024). Progesterone might help alleviate premenstrual syndrome symptoms through its diuretic and anxiolytic effects within the central nervous system, though current evidence is limited (Panay and Studd, 1997; Ford et al., 2012). Micronised progesterone can be administered orally (100 or 200 mg) or vaginally, with the latter route sometimes preferred for its ability to bypass first-pass metabolism in the liver, potentially enhancing tolerance.

Selective progesterone receptor modulators offer another treatment option for PMDD due to its antagonistic effects on progesterone receptors (Table 1). Mifepristone (RU 486), the first selective progesterone receptor modulators investigated, did not significantly improve symptoms of severe premenstrual syndrome (Chan et al., 1994). However, a second-generation selective progesterone receptor modulators, ulipristal acetate, is promising. Although primarily used as an over-the-counter emergency contraceptive, ulipristal acetate has been explored in PMDD treatment. In a proof-of-concept randomised controlled trial involving 95 women with PMDD, daily doses of 5 mg ulipristal acetate produced significant symptom improvement over 3 months compared to placebo, with a favourable safety profile (Comasco et al., 2021).

Stabilising the progesterone metabolite ALLO by inhibiting its conversion from progesterone via 5-alpha reductase may offer a promising approach to improving PMDD symptoms (Martinez et al., 2016) (Table 1). Modifying the formation of ALLO, which modulates the GABA system, represents an important avenue for developing a new treatment for PMDD (Schule et al., 2014; Szpunar and Freeman, 2021). Given the similarities in the hormonal underpinnings of both PND and PMDD, brexanolone–a synthetic form of ALLO treatment for PND (see Section 5 below) – may also serve as a novel therapeutic option for PMDD. Brexanolone enhances GABA-A receptor activity, stabilises dysfunctional GABA-A channels, and mimics ALLO, whose fluctuating levels during hormonal changes contribute to mood destabilisation (Edinoff et al., 2021). By restoring GABAergic function, brexanolone stabilises inhibitory neurotransmission, providing therapeutic relief from mood disturbances associated with hormonal fluctuations. Sepranolone, a negative modulator of the GABA-A receptor, offers another approach to PMDD treatment by inhibiting the effects of ALLO on the GABA-A system. Studies have shown that sepranolone significantly reduces PMDD symptoms (Bixo et al., 2017; Backstrom et al., 2021), providing an important contrast on how positive (brexanolone) and negative (sepranolone) GABA-A modulators can be effective in stabilising mood during hormonal fluctuations. However, Asarina Pharma has announced that it will no longer produce sepranolone due to financial difficulties (International Association for Premenstrual Disorder, 2024).

Another potential treatment avenue for PMDD involves targeting ALLO modulation. Dutasteride, a 5-alpha reductase inhibitor that prevents the conversion of progesterone into ALLO. While not yet widely studied or approved for PMDD, preliminary evidence suggests its promise. High-dose dutasteride (2.5 mg/day) has been shown to significantly reduce core PMDD symptoms such as irritability, sadness, anxiety, food cravings, and bloating compared to placebo (Martinez et al., 2016). However, low-dose dutasteride (0.5 mg/day) did not demonstrate a significant effect on PMDD symptoms when compared to placebo.

Progesterone, however, can exacerbate mood symptoms in susceptible women, as seen in those using progesterone-only contraceptives, such as progesterone-only pill and the levonorgestrel intrauterine device (Skovlund et al., 2016). Therefore, careful monitoring is essential. Additionally, a Cochrane review and meta-analysis on the use of progesterone for premenstrual syndrome found limited evidence supporting its effectiveness when used alone (Ford et al., 2012).

#### 2.1.4 Gonadotropin-releasing hormone (GnRH)

Gonadotropin-releasing hormone (GnRH) analogues are highly effective in treating severe PMDD (Wagner-Schuman et al., 2023) (Table 1). GnRH is a releasing hormone responsible for the release of follicle-stimulating hormone and luteinising hormone from the anterior pituitary. GnRH analogues are designed to ‘switch off’ the ovaries temporarily and chemically induce menopause. A meta-analysis of five clinical trials of GnRH for premenstrual syndrome concluded that GnRH are effective for reducing both physical and emotional premenstrual symptoms (Wyatt et al., 2004). Since then, further studies have shown that GnRH treatments are effective for PMDD (Pincus et al., 2011; Nguyen et al., 2017), with response rates reaching up to 75% compared to placebo (Nevatte et al., 2013).

Given that GnRH analogues fully suppress both progesterone and estrogen, the resulting estrogen deficiency often leads to significant side effects, particularly vasomotor symptoms and bone demineralisation (Nevatte et al., 2013). Consequently, GnRH analogues are typically reserved for severe cases of PMDD and limited to 6 months of use (Surrey, 1999). For extended treatment, add-back therapy with combination of estradiol and progestogen, or tibolone—an agent with estrogenic, androgenic and progestogenic effects—is required to mitigate the adverse effects of estrogen deficiency (Wyatt et al., 2004).

## 3 Postnatal depression (PND)

PND is a serious mood disorder affecting up to 17.22% of women globally following childbirth (Wang et al., 2021). It includes labile mood with pronounced anxiety and irritability, overwhelming feelings of inability to cope, confusion, early-onset insomnia, and diurnal variation in mood and energy levels. In very severe cases, suicide and thoughts of harming the baby can occur. PND can occur as a standalone depressive episode or as part of the bipolar spectrum, particularly in women with a history of bipolar disorder. If left untreated, PND can have profound and lasting negative effects on the parent, child, and family as a whole (Myers and Johns, 2018). Children of mothers with PND are particularly vulnerable, facing an increased risk of cognitive, emotional, and developmental delays, as well as verbal deficits and impaired social skills later in life (Brand and Brennan, 2009; Slomian et al., 2019; Saharoy et al., 2023).

During pregnancy and the *postpartum* period, there are many significant fluctuations in estrogen, progesterone, testosterone, corticotropic-releasing hormone, and cortisol–which impact brain chemistry and neural circuits (Schiller et al., 2015). This is compounded by the stress and physical demands of caring for a newborn. ALLO increases approximately 40-fold in serum during pregnancy (Luisi et al., 2000; Paoletti et al., 2006), enhancing GABA-A receptor signalling with significant inhibitory effects leading to anxiolytic and sedative properties. Studies in rodents indicate that after delivery ALLO levels drop sharply, restoring pre-pregnancy brain steroid chemistry and initiating changes related to lactation (Brunton et al., 2009). Imaging studies of women with PND have shown heightened ALLO and monoamine oxidase activity, along with reduced serotonin activity levels (Epperson et al., 2006). These findings suggest that disruptions in the typical *postpartum* regulation of ALLO may contribute to the development of depressive symptoms.

### 3.1 Hormone treatments for PND

Current treatments for PND often focus on psychotherapies and antidepressants, especially SSRIs. Electroconvulsive therapy may also be considered for severely affected women who do not respond to other treatments. However, these standard approaches tend to overlook the significant endocrine shifts that occur during pregnancy and immediately *postpartum*.

Hormonal treatments are an emerging area of interest for addressing PND (Table 2), although they remain significantly understudied compared to traditional treatments. Some studies suggest that postnatal estrogen treatment might help stabilise mood in affected women. For example, a double-blind randomised controlled trial by Gregoire et al. found that transdermal estrogen, combined with cyclical progesterone, was modestly more effective than placebo in reducing symptoms of moderate to severe PND (Gregoire et al., 1996). However, larger studies are needed to substantiate estrogen’s efficacy in this context and to establish safe protocols for its use in the *postpartum* period.

**TABLE 2:** Summary of hormone treatment options for PND.

Drug type	Drug name	Dosage	Use recommendation	Findings/comments
Estradiol	Estrogen	Transdermal 200 μg daily	Take in combination with cyclical progesterone (dydrogesterone)	Modestly more effective than placebo in reducing symptoms of moderate to severe PND (Gregoire et al., 1996)
Synthetic allopregnanolone	Brexanolone	Infusion 30 μg/kg per h (0–4 h); 60 μg/kg per h (4–24 h); 90 μg/kg per h (24–52 h); 60 μg/kg per h (52–56 h); 30 μg/kg per h (56–60 h)	Single continuous intravenous infusion for 60 h	Rapid relief of PND symptoms with 60 h of administration (Kanes et al., 2017; Meltzer-Brody et al., 2018); limited by high cost and requirement for inpatient administration
Allopregnanolone	Zuranolone	50 mg daily	14-day oral treatment	Reduced depressive symptoms in women with PND (Deligiannidis et al., 2023); limited accessibility due to high cost

Recent advances in the understanding of the role of neuroactive steroids, particularly ALLO, in mood regulation have led to the development of new hormonal treatments specially for PND. The FDA-approved brexanolone was the first allopregnanolone medication specifically approved for PND (Table 2). Phase II (Kanes et al., 2017) and III (Meltzer-Brody et al., 2018) clinical trials have demonstrated that brexanolone rapidly reduces PND symptoms within 60 h of administration. By acting as a positive allosteric modulator of GABA-A receptors, brexanolone provides rapid symptom relief in PND. Although, its high cost and requirement for inpatient administration present challenges, especially for new mothers.

A recent oral ALLO medication, zuranolone, has also been approved by the FDA (Table 2). This 14-day oral treatment offers a less invasive approach than brexanolone and has shown efficacy in reducing depressive symptoms in women with PND (Deligiannidis et al., 2023). However, the high cost of zuranolone limits its accessibility.

## 4 Menopausal depression

Perimenopause marks the transitional phase from a woman’s reproductive years to menopause, typically occurring between the ages of 42 and 52. This stage is clinically identified by irregular menstrual cycles or variations in cycle duration. According to the Stages of Reproductive Aging Workshop (STRAW) criteria (Soules et al., 2001), perimenopause is indicated when cycle lengths vary by at least 7 days, with full menopause confirmed after a year without menstruation.

During the menopausal transition, which can span 8–10 years, significant shifts occur in gonadal hormones, including estrogen, progesterone, testosterone, and their precursors (Herson and Kulkarni, 2022). These hormonal fluctuations influence the central nervous system and have been shown to disrupt serotonin receptor expression, which is critical for maintaining serotoninergic system function (Freeman et al., 2006; Barth et al., 2015). Estrogen modulates serotonin receptor density, binding, and transport in key brain regions such as the prefrontal cortex and hippocampus (Wallace et al., 2006; Tuscher et al., 2016), and its decline during menopause is associated with impaired serotonin signalling (Hwang et al., 2020). This disruption also has downstream effects on the dopaminergic system, which relies on balanced serotonergic input for optimal function (Almey et al., 2015; Niederkofler et al., 2015). Such neurochemical instability contributes to the development of depression, irritability, and anxiety, while reducing estrogen’s neuroprotective effects, which in turn may lead to cognitive decline and emotional dysregulation in postmenopausal women (Dumas et al., 2012; Newhouse and Dumas, 2015).

Additionally, lower levels of dehydroepiandrosterone sulfate, an adrenal precursor to estrogen and a regulator of serotonergic and GABA signalling, is also associated with increased depression in perimenopausal and postmenopausal women (Barrett-Connor et al., 1999; Herson and Kulkarni, 2022). Studies show that lower dehydroepiandrosterone sulfate levels correlate with greater depressive symptoms (Morrison et al., 2001; Schmidt et al., 2002), likely compounding the effects of fluctuation estrogen levels.

Depression during the menopause transition differs from a typical major depressive disorder. ‘Menopausal depression’ is often marked by persistent anxiety, irritability, anger, intense bouts of sadness and difficulties with concentration and memory, and a diminished interest in daily activities (Kulkarni et al., 2018a; Kulkarni et al., 2024). Unlike standard major depressive disorder, menopausal depression appears to stem largely from hormonal fluctuations in the central nervous system during menopause (Herson and Kulkarni, 2022). Notably, these central nervous system changes can begin up to 5 years before physical symptoms of menopause appear, making them easy to overlook. This often-silent onset increases the risk of unrecognised depressive symptoms, with approximately 40% of women experiencing depression, compared to lower rates in premenopausal women (Badawy et al., 2024).

### 4.1 Hormone treatments for menopausal depression

Current guidelines from the Australian, North American and International Menopause Societies recommend antidepressants, psychological therapy, and lifestyle changes as primary treatments for depression in perimenopausal and menopausal women (Stuenkel et al., 2015). However, these treatments often yield suboptimal outcomes in this group (Maki et al., 2019). Antidepressants, particularly SSRIs like escitalopram, are ineffective for many women (Soares et al., 2011), with some older women developing tachyphylaxis (Grigoriadis et al., 2003). Studies indicate that SSRIs may be less effective after menopause in women who do not receive hormone replacement therapy (HRT) (Parry, 2010). Conversely, adding estrogen to SSRIs has been shown to accelerate the antidepressant effect and increase its efficacy (Schneider et al., 1997; Nagata et al., 2005; Zanardi et al., 2007). Desvenlafaxine 50 mg/day has shown some efficacy in menopausal women with major depressive disorder (Kornstein et al., 2014), while sertraline has demonstrated effectiveness and modest cognitive improvement in postmenopausal women (Rasgon et al., 2007; Parry, 2010). Yet, clinical guidelines still recommend SSRIs as first-line treatments, often overlooking the potential benefits of hormone therapy. Recognising this challenge, the 2019 NICE guideline (NICE, 2015) advises clinicians to “consider HRT to alleviate low mood that arises as a result of the menopause.” SSRIs also have several side effects, including serotonin syndrome, agitation, nausea, decreased libido, and emotional numbing, which can impair women’s quality of life. For women with menopause-related insomnia, irritability, and anxiety, fluoxetine may even worsen these symptoms (Kulkarni, 2018).

#### 4.1.1 Menopause hormone therapy (MHT)

Menopause hormone therapy (MHT), previously known asHRT, has long been a key treatment for managing physical menopausal issues, such as vasomotor symptoms. However, MHT has not been recommended in menopause guidelines for the treatment of menopausal depression.

There are many types of MHT, which include different types of estrogens and progestogens in differing doses (Table 3). A 4-year study reported that conjugated equine estrogen (0.45 mg/day, with cyclic progesterone) improved some depressive symptoms compared to placebo (Gleason et al., 2015). However, a large 4-month trial investigating the effects of conjugated equine estrogen (0.625 mg/day, with continuous medroxyprogesterone acetate) found no significant impact on mood or emotional wellbeing, suggesting that the formulation and dosing regimen may influence the therapeutic efficacy of MHT for affective symptoms (Maki et al., 2007).

**TABLE 3:** Summary of hormone treatment options for menopausal depression.

Drug type	Drug name	Dosage	Use recommendation	Findings/comments
Estradiol	Conjugated equine estrogen	0.45–0.625 mg daily	Take in combination with cyclic progesterone	Improved some depressive symptoms in combination with cyclic progesterone when compared with placebo (Gleason et al., 2015); no effect on affect in combination with continuous medroxyprogesterone acetate (Maki et al., 2007)
	Estrogen	Transdermal 100–500 µg daily	May take as combined therapy with traditional antidepressant	Significant improvement with depressive symptoms in perimenopausal women (Schmidt et al., 2000; Soares et al., 2001); more effective if taken as combination with antidepressant than if taken alone (Schneider et al., 1997; Nagata et al., 2005)
Second-generation selective estrogen receptor modulator (SERM)	Raloxifene	60 mg daily	Trialled for menopausal depression	No significant benefit over placebo for menopausal depression (Schmidt et al., 2021)
Third-generation SERM	Bazedoxifene	10–40 mg daily	Combined with 0.45 or 0.625 mg conjugated estrogen	Reduces vasomotor symptoms, prevents bone loss, improves sleep quality, enhances quality of life for menopausal women (Taylor and Ohleth, 2012; Mirkin et al., 2014; Lello et al., 2017)
Selective tissue estrogenic activity regulator	Tibolone	2.5 mg daily	For menopausal depression	Effective in reducing depressive symptoms in menopausal women (Kulkarni et al., 2018b)
Testosterone	Methyltestosterone	1.25–5 mg daily	Non-oral routes preferred because of a neutral lipid profile	Insufficient evidence for menopausal symptoms other than low libido (Islam et al., 2019)
	Testosterone	Patch 150–300 µg daily, transdermal gel 300 µg daily, transdermal cream 10 mg daily, or spray 56 μL, 90 μL, or 180 µL daily		
	Dihydrotestosterone (androstanolone)	2.5 mg twice daily		
	Testosterone enanthate	Intramuscular injection 3 mg, 6.25 mg, 12.5 mg, or 25 mg weekly		
	Micronised testosterone	Sublingual 1.25 mg twice daily		

One small study showed that perimenopausal women with minor and major depression treated with transdermal estradiol (0.5 mg/day) improved significantly after 3 weeks, compared to women receiving placebo (Schmidt et al., 2000). In a secondary analysis, both groups received an additional 3 weeks of transdermal estradiol 0.5 mg/day. By the end of the full 6 weeks, the treatment group sustained their reduced depression scores relative to baseline, while the placebo patients also showed significantly improved scores following the active treatment phase (Schmidt et al., 2000).

In a double-blind clinical trial by Soares et al. (2001), transdermal estradiol (100 μg/day) was administered to perimenopausal women with endocrinologically confirmed diagnoses. The study demonstrated a significant reduction in depressive symptoms in the estrogen group compared with placebo, over a 12-week treatment period, with most participants achieving complete remission and experiencing similar side effects to the placebo group (Soares et al., 2001).

Additionally, other studies indicate that combined therapy with estrogen and traditional antidepressants is more effective in treating menopausal depression than either treatment alone (Schneider et al., 1997; Nagata et al., 2005). This synergistic effect may result from estrogen’s ability to modulate serotonergic and dopaminergic systems, enhancing the efficiency of antidepressants.

#### 4.1.2 Selective estrogen receptor modulators (SERMs)

Extended use of estrogen and progestins raises potential concerns regarding the impact on breast and uterine tissue, prompting interest in selective estrogen receptor modulators (SERMs) as an alternative. SERMs offer many of estrogen’s therapeutic benefits–supporting bone health, improving lipid levels, and potentially benefiting cognitive function–while minimising risks to the breast and uterus (Dutertre and Smith, 2000; Littleton-Kearney et al., 2002). Initially developed to treat breast cancer and osteoporosis (Komm and Mirkin, 2014), SERMs may have potential as additional treatment for menopause depression (Table 3).

Raloxifene, a second-generation SERM, has been established as a safe and effective treatment for postmenopausal women, particularly in supporting bone health (Komm and Mirkin, 2014). It may also affect neuronal tissue by modulating serotonin receptors, blocking estrogen activation of estrogen response elements on DNA, and promoting brain-derived neurotrophic factor (Littleton-Kearney et al., 2002; Bourque et al., 2014; Ishihara et al., 2015). Research by Kulkarni et al. found that high doses of raloxifene (120 mg/day) improved clinical outcomes in treatment-resistant schizophrenia (Kulkarni et al., 2016). However, evidence for raloxifene’s effects on depression remains limited (Yang et al., 2013). In a recent 8-week randomised controlled trial, Schmidt et al. found no significant benefit of raloxifene over placebo in treating menopausal depression (Schmidt et al., 2021), and similar studies have reported minimal impact on depressive symptoms (Khorsand et al., 2018).

Bazedoxifene, a third-generation SERM, has a unique pharmacological profile that may be particularly well-suited for managing menopausal symptoms, with a potentially safer breast tissue profile favourable for long-term use (Pickar and Komm, 2015). When combined with conjugated estrogens, bazedoxifene effectively reduces vasomotor symptoms, prevents bone loss, improves sleep quality, and enhances the quality of life for menopausal women (Taylor and Ohleth, 2012; Mirkin et al., 2014; Lello et al., 2017). These benefits have been shown in five Phase III randomised controlled trials (Pickar et al., 2009; Pinkerton et al., 2009; Kagan et al., 2010; Skouby et al., 2015; Umland et al., 2016). However, its potential role in addressing mental health concerns related to menopause is still being explored. There is an ongoing double-blind randomised controlled trials of bazedoxifene plus conjugated estrogen evaluating its efficacy in menopausal depression (ACTRN12620001015932).

Tibolone, is a synthetic steroid that is classified as a selective tissue estrogenic activity regulator. Tibolone is also emerging as a promising treatment for menopausal depression due to its potential neuroprotective effects, which stem from antioxidant activity at the cellular level (Cardona-Gomez et al., 2001; Genazzani et al., 2006; Pinto-Almazán et al., 2017; Del Rio et al., 2020). In a 6-month study involving women following surgical menopause, tibolone and transdermal estradiol significantly improved menopausal, depression, and anxiety scores compared to placebo (Baksu et al., 2005). A recent 12-week randomised controlled trials further showed tibolone’s effectiveness in reducing depressive symptoms in menopausal women without significant side effects (Kulkarni et al., 2018b). However, findings in postmenopausal women are mixed, with some studies indicating that tibolone may not consistently outperform placebo in treating depression (Lam et al., 2004; Karşıdağ et al., 2012; Kim et al., 2019), and may not enhance the antidepressant effects of SSRIs (Schneider et al., 1997; Berlanga et al., 2004). Unlike tamoxifen and raloxifene, tibolone effectively reduces vasomotor symptoms and vaginal dryness, with fewer bleeding irregularities than traditional MHT (Hammar et al., 1998), due to estrogen, progesterone, and androgen metabolites, which support endometrial and mammary tissue health, maintain bone density, and provide relief from vaginal dryness (Kloosterboer, 2004).

#### 4.1.3 Testosterone

Testosterone therapy is gaining attention as a potential treatment for cognitive impairment and mood disturbances in menopausal women (Table 3). Although often viewed as a ‘male’ hormone, testosterone plays a key role in women’s physiology, including mood regulation, energy levels, libido, and cognitive function (Scott and Newson, 2020). In women, testosterone levels decline with age and can be as much as 50% lower in menopausal women compared with younger women (Davis, 2023), which may contribute to symptoms like brain fog and depression (Glynne et al., 2024). However, studies have indicated that during the menopause transition, phases where there is a higher testosterone-to-estradiol ratio may be linked to increased depressive symptoms (Sander et al., 2021).

The current evidence for testosterone therapy treating menopausal depression is still emerging. An early randomised controlled trials found that testosterone, whether used alone or added to MHT, enhanced mood, energy, and libido in women with surgical menopause beyond what estrogen therapy alone provided (Sherwin and Gelfand, 1985). In a study of 978 perimenopausal and postmenopausal women, Riesel et al. reported that MHT, with or without testosterone, led to significant improvement in menopause-related symptoms over 3 months, with ‘profound low mood’ showing the greatest improvement (Reisel et al., 2024). For women already using standard MHT, the addition of transdermal testosterone improved concentration, memory, mood and motivation, with the most notable improvements seen in mood and motivation (Kamal et al., 2023). Similarly, a recent pilot study found that 4 months of transdermal testosterone treatment significantly improved mood and cognition, with mood improving more than cognition (47% vs 39%) (Glynne et al., 2024). However, a meta-analysis concluded that randomised controlled trials evidence supporting testosterone therapy for menopausal symptoms other than low libido is insufficient (Islam et al., 2019). Concerns about testosterone treatment in postmenopausal women primarily focus on adverse reactions such as hirsutism and acne (Braunstein, 2007; Islam et al., 2019). These side effects are generally dose- and time-dependent and typically reversible upon discontinuation of testosterone therapy. Nonetheless, more research is needed to determine the efficacy of testosterone, specifically for depressive symptoms during menopause. Furthermore, long-term prospective studies are essential to gather comprehensive data on the safety profile of testosterone use in women, as the majority of current evidence is limited to a maximum duration of 2 years (Braunstein, 2007).

## 5 Clinical suggestions and future direction

For PMDD, treatment typically focuses on reducing sensitivity to luteal phase fluctuations in estrogen and progesterone. Continuous COCPs with drospirenone are used for cyclical gonadal hormone stabilisation (Kelly et al., 2010), but newer COCPs such as nomegestrol acetate (2.5 mg) with 17-beta estradiol (1.5 mg) appear to have better outcomes (Robertson et al., 2021), particularly for women who have developed depressive symptoms when taking other hormone con9traceptives (Mu and Kulkarni, 2022). Combining intermittent use of SSRIs (taken in the luteal phase) with a COCP is a useful treatment. If not completely effective, the SSRI could be increased to daily use in conjunction with COCP (Kulkarni, 2022). In more persistent and difficult to treat cases of PMDD, GnRH analogues may induce a temporary menopausal state to halt hormone cycles, with add-back estradiol to prevent hypoestrogenism-related bone density loss (Wyatt et al., 2004).

In treating PND, the sharp *postpartum* estrogen drop is a contributing factor, especially in hormone-sensitive women. Recent causal theories for PND include altered allopregnanolone ‘switch’ mechanisms. Treatment with newer allopregnanolone agents appears promising for the future. Transdermal estradiol can stabilise mood and relieve depressive symptoms, and may be used in combination with SSRIs for comprehensive mood and anxiety support. For breastfeeding mothers, lower estrogen doses minimise lactation interference (Jin et al., 2024).

In menopausal depression, the major symptoms appear to be caused by inherent sensitivity to the big fluctuations in the central nervous system of the gonadal steroids. The fluctuating impact on many neurochemical and neurocircuitry systems appears to result in significant anxiety, depression, memory and concentration challenges that in combination with vasomotor body symptoms - all impair the woman’s quality of life. Menopause hormone treatments such as transdermal estradiol with micronised progesterone effectively address both depressive and vasomotor symptoms.

Tailored dosing strategies can improve treatment efficacy. Luteal-phase only dosing may be appropriate for PMDD (Freeman, 2004), while continuous dosing better suits PND and menopausal depression. Transdermal delivery is often preferred over oral due to lower thromboembolic risk and stable hormone levels (Scarabin, 2018; Vinogradova et al., 2019). Both oral and transdermal estrogen administration demonstrate similar benefits regarding improving bone density, glucose metabolism, and lipid profiles, as well as comparable risks related to breast cancer, endometrial disease, and cardiovascular outcomes in postmenopausal women (Goldstajn et al., 2023). Educating patients and involving them in decision-marking helps ensure empowerment and adherence, with regular re-evaluation to accommodate changing health needs.

Full physical assessments for patients with histories of hormone sensitive cancers, thromboembolism, or cardiovascular concerns is crucial before commencing hormone therapy. In general, SERMs and selective tissue estrogenic activity regulator can serve as alternatives when hormone therapy is contraindicated. Cardiovascular and metabolic biomarkers, such as blood pressure and lipid levels, should be monitored, especially for menopausal women using estradiol. For PMDD patients taking GnRH analogues, bone density monitoring and supplementation with calcium and vitamin D are advised to counter osteoporosis risks.

Gonadal hormones are potent neurosteroids and have many complex and interrelated effects on brain chemistry and circuitry. Some women are particularly sensitive to hormone fluctuations and hence respond to monthly cycle changes with developing PMDD, or have significant postnatal or perimenopausal depression. Each woman who experiences mental ill health as a result of gonadal hormone fluctuations needs a careful, holistic and collaborative approach to treatment–so that she can achieve optimal outcomes. Hormone therapies provide another group of possible treatments, but more clinical trials are required to enable treatment guidelines to understand and guide the best consideration of the type and dose of gonadal hormone treatment, with each woman receiving tailored treatment.

## 6 Conclusion

In conclusion, gonadal hormone therapies offer promising options for managing PMDD, PND and menopausal depression by addressing hormonal imbalances that contribute to mood fluctuations. However, the variability in individual responses highlights the need for personalised treatment plans, considering factors such as hormone type, dosage, and administration route. Exciting new hormone treatments are emerging, which may offer more targeted and effective solutions. However, more research is needed, particularly to evaluate these newer therapies. To strengthen the evidence base, large-scale randomised controlled trials are essential, as many existing studies are small. Addressing these research gaps will help optimise treatment approaches and improve the quality of life for women affected by these conditions.
